# Neuropsychological deficits in patients with favorable outcomes after middle cerebral artery infarction treated with thrombectomy

**DOI:** 10.3389/fneur.2026.1686604

**Published:** 2026-02-04

**Authors:** Hannah Hild, Christian Foerch, Ariane Martinez Oeckel

**Affiliations:** 1 Department of Neurology, Goethe University, Frankfurt am Main, Germany; 2 Department of Neurology, RKH Klinikum Ludwigsburg, Ludwigsburg, Germany

**Keywords:** functional outcome, ischemic stroke, modified Rankin Scale, neuropsychological deficits, quality of life

## Abstract

**Introduction:**

Functional outcomes following stroke thrombectomy are commonly measured by the modified Rankin Scale (mRS). Due to hemisphere-specific representation of neuropsychological function, the mRS might underestimate persisting functional deficits, particularly in large right-hemispheric strokes. This study aimed to assess infarct volumes and neuropsychological symptoms in patients with left- and right-sided middle cerebral artery (MCA) infarctions treated with thrombectomy, having a favorable outcome at 3 months. We hypothesized that patients with right-sided strokes are affected by larger infarct volumes and suffer from neuropsychological and cognitive impairment despite being classified as functionally recovered on the mRS.

**Methods:**

The study cohort consisted of 105 patients with MCA infarctions treated with thrombectomy and a favorable outcome at 3 months after stroke according to mRS 0-1. The infarct volumes after thrombectomy were measured using imaging data. Consenting patients received follow-up examinations through telephone interviews and written questionnaires (Stroke Impact Scale-16, Short Form-36). Patients with the largest infarct volumes were additionally assessed with a neuropsychological test battery (Test of Attentional Performance – Mobility-Version, TAP-M) and the Montreal Cognitive Assessment.

**Results:**

The mean infarct volume was larger in patients with right-sided (23.3 mL) strokes compared to left-sided (8.5 mL) strokes (*p* < 0.05). Loss of working ability was reported by 24.3% of patients with right MCA infarctions and 2.9% of patients with left MCA infarctions (*p* < 0.05). Compared to age-dependent standard values, patients with right-sided MCA infarctions showed longer reaction times, impaired divided attention, and subclinical neglect to the left. Additionally, the majority of these patients showed mild cognitive impairment.

**Discussion and conclusion:**

These findings indicate that patients with right MCA infarctions, being categorized as having a “favorable functional outcome” on the mRS, suffer from relevant neuropsychological deficits, subclinical neglect, and reduced quality of life.

## Introduction

### Background

Stroke affects more than 12.2 million people globally per year, with a relatively balanced distribution between strokes of the right and left hemispheres (1, 2). The modified Rankin Scale (mRS) is the most frequently used disability scale to assess clinical outcomes after stroke (3) and to determine treatment effectiveness (4, 5). The mRS score ranges from 0 to 6, measuring the degree of disability or dependence in activities of daily life, with grade 0 corresponding to no symptoms, grade 5 to severe disability, and grade 6 to death (4). The mRS focuses on motor outcomes and is better suited to assess deficits following lesions of the dominant hemisphere (6). In particular, neuropsychological, cognitive, and emotional impairment, pain, and the ability to work and pursue hobbies are not measured by the mRS. Thus, the mRS reflects a patient’s overall outcome only to a limited extent (7, 8).

The actual condition of the patient and the patient’s functional deficit according to the mRS can be distorted (9, 10), especially for right-hemispheric strokes. Right-hemispheric strokes often result in subclinical deficits, which are more difficult to perceive, e.g., neglect and other specific cognitive disabilities (6, 8), and thus score low on the mRS. The overall functional outcome is at least as poor in right-hemispheric stroke patients as in left-hemispheric stroke patients (11), as they suffer from relevant neuropsychological and cognitive impairment and reduced quality of life (7).

### Objective

We aimed to assess the clinical and demographic characteristics of patients with left- and right-hemispheric strokes who underwent thrombectomy and achieved a favorable outcome (mRS 0-1) at 3 months. This study aimed to assess whether patients with favorable outcomes on the mRS have larger stroke volumes in right-sided strokes compared to left-sided strokes. We hypothesized that patients with large right-hemispheric strokes exhibit neuropsychological and cognitive deficits on assessment despite being classified as functionally recovered on the mRS.

## Methods

### Data source and study population

The study was approved by the Local Ethics Committee of the University Hospital Frankfurt am Main, Germany (reference number 20-1070). This analysis used data from the German Stroke Registry, collected at the Department of Neurology, University Hospital Frankfurt am Main. The German Stroke Registry (GSR) prospectively enrolled all consecutive stroke patients who underwent endovascular therapy. For follow-up, the mRS was assessed at 3 months post-stroke via telephone by clinical study investigators from the Department for Neurology. For further follow-up, patients’ long-term psychological performance was assessed by telephone between June 2021 and October 2021, and in person between May 2022 and June 2022, by the author HH. For the present analysis, we selected all patients from this database admitted between March 2016 and November 2020 who met the following inclusion criteria: (i) first-time stroke, (ii) acute ischemic stroke of the left or right middle cerebral artery territory, (iii) endovascular therapy (mechanical thrombectomy), (iv) age ≥18 years, and (v) modified Rankin Scale (mRS) score of 0 or 1 at 3 months post-stroke. The exclusion criteria included (i) bilateral stroke and (ii) multi-territory stroke.

### Outcomes

The primary study outcome was the stroke lesion volume, with age and sex as covariates. The stroke volume was measured independently by two researchers (HH and AMO) using magnetic resonance imaging (MRI) and computed tomography (CT) data acquired at a mean of 1.8 days (range: 0–11 days; median 1 day) after thrombectomy. Primarily, non-contrast CT data were used for stroke volume measurement (98 patients). If a CT scan was unavailable, diffusion-weighted or fluid-attenuated inversion recovery MR imaging was used instead (7 patients). Following thrombectomy, one patient experienced such a substantial hemorrhagic transformation that precise measurement of the ischemic infarct area was not feasible. Consequently, stroke volume was measured using the latest CT scan after complete resorption of blood (266 days post-thrombectomy). To calculate infarct volume, a manual delineation approach was used. The affected brain region was manually delineated on each axial imaging slice using the open-source software ImageJ, and the corresponding area (in mm^2^) was automatically calculated. Subsequently, the measured lesion area on each slice was multiplied by the respective imaging slice thickness (5 mm for all scans) and converted to milliliters. This method allowed precise measurement in the presence of variable lesion shapes and multiple lesions. The results of the volumetric analysis obtained independently by both researchers were averaged, and the final stroke volume was indicated in milliliters (mean variance between independent measurements 0.7 mL; median 0.3 mL; range: 0–2.8 mL).

The secondary study outcome was the patient’s neuropsychological performance at follow-up. Fully written, informed consent was obtained from all subjects. In case of severe aphasia, detailed information was given to the patients’ relatives or legal guardians. All patients were contacted via telephone by the same interviewer, and, if necessary, additional information was provided by relatives. During the telephone interview, the current functional outcome (mRS) was assessed, and all patients were asked to answer the following questions in the German language: (1) Were you able to return to your workplace after your stroke, and do you still work there? (2) What hobbies do you have, and have you changed them after your stroke? (3) Are you still able to drive a car? (4) Did you suffer a recurrent stroke? (5) Do you have any other medical issues, such as chronic heart disease or psychological problems? The first three questions were designed to identify crucial changes in the quality of life and psychological health post-stroke, including regaining autonomy, engaging in hobbies and work, and participating in activities that provide a sense of normalcy similar to their pre-stroke experiences (12, 13). Questions 4 and 5 were designed to assess the study demographics and potential bias in patient follow-up. These questions have not yet been validated in other studies.

Patients who consented then received two validated questionnaires by mail: the Short Form-36 (SF-36) and the Stroke Impact Scale-16 (SIS-16). The SF-36 questionnaire consists of 36 questions assessing general health and quality of life and has been shown to be valid in stroke patients for measuring mental and physical health, including mild functional loss relevant to independent living (14). SF-36 scores are normalized for age and gender in the German population. The following eight subsections—vitality, physical functioning, bodily pain, general health perceptions, physical role functioning, emotional role functioning, social role functioning, and mental health—can be grouped into two summary dimensions: the physical and mental component summary (15). The SIS-16 questionnaire consists of 16 questions assigned to the four categories of mobility, strength, hand function, and activities of daily living to evaluate physical functions specifically after stroke. The SIS-16 is sensitive for identifying a wide range of deficits and can be used to distinguish low-level disabilities (16, 17).

Finally, we assessed whether larger stroke lesion volumes translate into persistent and functionally relevant neuropsychological deficits. For doing so, we performed a detailed neuropsychological assessment on those 25% of patients having the largest stroke lesion volumes (cutoff: 18 mL, *n* = 15). This threshold was selected based on previous studies indicating that infarct volumes exceeding 15–20 mL are associated with a marked decline in functional outcomes, and, thus, our 18 mL threshold reflects an evidence-based distinction between small and medium-to-large infarcts (18–20). The Montreal Cognitive Assessment (MoCA) Test was performed in person using pencil and paper, and standardized instructions were given. Furthermore, patients were assessed for approximately 40 min based on the mobility version of the “Test of Attentional Performance” (TAP-M), including the five subtests: alertness, Go/NoGo, divided attention, active visual field, and visual scanning. TAP-M is a standardized software package that includes tasks that consist of simple and easily distinguishable stimuli to which patients respond through a simple motor reaction. The subtests facilitate the evaluation of a spectrum of visuospatial, non-spatial, and executive attentional aspects. These components include alertness, divided attention, flexibility of focused attention, inhibitory processes, working memory, visual search, selective visual attention, and suppressing potentially distracting stimulation (21).

### Statistical analysis

To ensure the statistical robustness of our study, a sample size calculation using the website clincalc.com was conducted based on the primary outcome of the stroke lesion volume. The calculation was based on an expected mean lesion volume of 30 ± 20 mL in left MCA stroke and 40 mL in right MCA stroke. These estimates are consistent with previously published lesion volume ranges following mechanical thrombectomy in MCA strokes (22). Assumptions for the sample size calculation included an expected significance level of *α* = 0.05 and a power of 1−*β* = 0.8. The sample size calculation indicated that a total of 108 participants would be required. The same tools and assumptions were used to calculate the sample size for the secondary outcome, the Stroke Impact Scale-16 (SIS-16). Reference values for the left-hemispheric stroke group (with favorable outcomes, mRS 0-1) were derived from Duncan et al. (23), reporting a mean SIS-16 score of 88 ± 10 points. Based on clinically relevant differences, we assumed a reduced SIS-16 score of 75–80 points for patients with right-hemispheric infarctions. Using these estimates, the required sample size was determined to be between 18 and 50 participants per group, depending on the expected effect size.

Under the assumption of normal distribution of SIS-16 scores, an independent two-sided *t*-test was used to compare the two groups (left vs. right MCA stroke) regarding post-stroke physical function as assessed by SIS-16.

Patient characteristics were described using numbers and proportions for categorical data, and mean ± standard deviation as well as first, second, and third quartiles for continuous data. Patient characteristics included age, sex, modified Rankin Scale score at three time points (discharge, 3 months, and follow-up), and admission year and month. Stroke volume was compared between two patient groups with stroke in the left versus right hemisphere using the Mann–Whitney *U* test. The ability to work and drive, as well as the maintenance of hobbies, was compared between right- and left-hemispheric strokes using a Pearson chi-squared test. TAP-M subtest results were automatically produced by the software and included age-standardized (18–89 years) T-norms for median reaction time, errors, and omissions. To assess whether patients with stroke deviated from normative values, we compared T-scores from each patient group (right vs. left MCA stroke) against the normative mean (*T* = 50) using one-sample, one-tailed *t*-tests.

To address potential confounding effects of age and follow-up duration, additional multivariate analyses (for SIS-16 and SF-36; *n* = 53) and logistic regression (for MoCA and working ability) analyses were performed. Age at stroke was dichotomized using a median split (<66 vs. ≥66 years), and follow-up duration was categorized as short-term (≤3 years) and long-term (>3 years) to ensure sufficient group sizes. Both variables were included as covariates in the respective models.

## Results

### Cohort description

Of a total of 408 patients listed in the German Stroke Registry between 2016 and 2020, 122 patients met the inclusion criteria for the present analysis. However, 17 patients met the exclusion criteria due to the presence of bihemispheric or non-MCA strokes and were subsequently excluded from the study. Thus, a total of 105 patients were included in this study. The mean age was 64.3 ± 14.1 years, and 42% were female. Right-hemispheric strokes were present in 52% of patients. The mean stroke volume was 23.3 mL ± 39.9 mL in patients with right MCA infarction and 8.5 mL ± 12.1 mL in patients with left MCA infarction (*p* = 0.04), as shown in Table 1.

**Table 1:** Demographics.

Characteristic	Left-sided stroke	Right-sided stroke
**Total, *n* (%)**	50 (100.0)	55 (100.0)
**Sex, *n* (%)**		
Male	31 (62.0)	30 (54.5)
Female	19 (38.0)	25 (45.5)
**Age at stroke [years]**		
Mean (SD)	65.3 (15.1)	63.5 (13.4)
Median (p25, p75)	67.2 (53.6, 76.3)	65.0 (55.9, 73.0)
Minimum, maximum	25, 95	26, 90
**Stroke volume [milliliter]**		
Mean (SD)	8.5 (12.1)	23.3 (39.9)
Median (p25, p75)	4.9 (0.4, 9.9)	7.7 (0.7, 29.1)
Minimum, maximum	0, 47	0, 243
**Modified Rankin Scale (mRS)**		
At discharge		
Median (p25, p75)	1 (0, 2)	1 (0, 1)
Minimum, maximum	0, 5	0, 4
At 90-day follow-up		
Median (p25, p75)	1 (0, 1)	0 (0, 1)
Minimum, maximum	0, 1	0, 1
At telephone follow-up		
Median (p25, p75)	1 (1, 3)	1 (0, 1)
Minimum, maximum	0, 6	0, 6

### Follow-up

**Figure 1:**
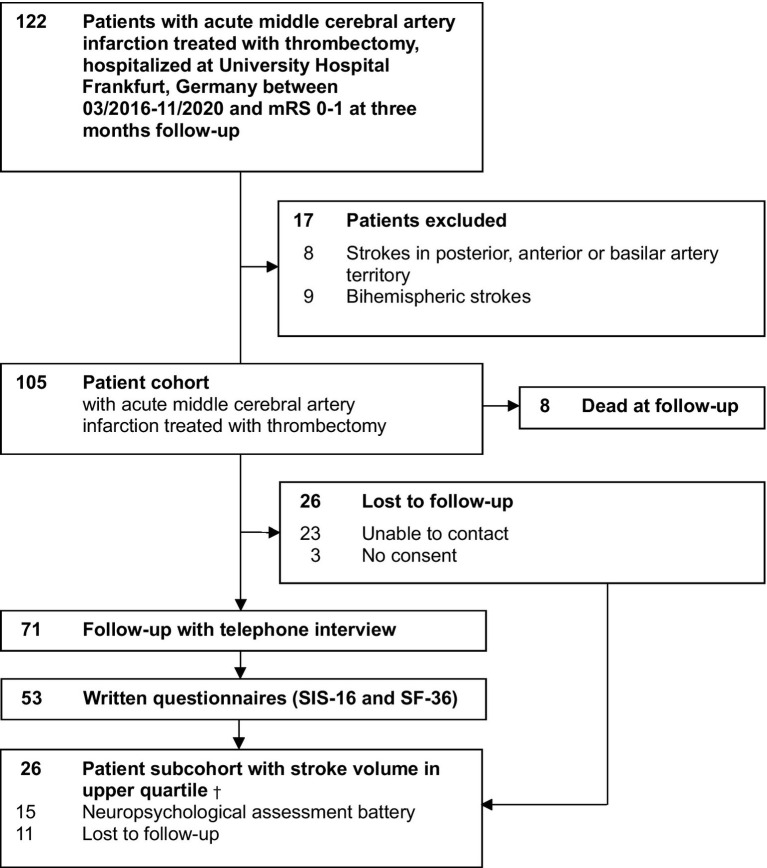
Ascertainment of the cohort. mRS, Modified Rankin Scale; SIS-16, Stroke Impact Scale-16; SF-36, Short Form-36. †Subcohort selected from the total patient cohort (*n* = 105).

Patients were contacted between 1 and 4 (mean 2) years after stroke by telephone, and if they consented, telephone interviews and postal questionnaires were added. During the following year, neuropsychological testing in person was completed. A total of 71 patients agreed to participate in the telephone interview, while 26 patients (25%) were lost to follow-up (3% declined to consent, and 22% were unable to be contacted), and 8% had already passed away at the time of contact, as shown in Figure 1. The proportion of patients lost to follow-up did not differ between those with left- vs. right-hemispheric strokes. In the remaining 71 follow-up patients, the mean age at stroke symptom onset was 63.8 ± 15.3 years in patients with left-hemispheric stroke (*n* = 34) and 64.8 ± 12.8 years in patients with right-hemispheric stroke (*n* = 37). Stroke lesion volume in this cohort was 12.0 mL in patients with left-sided stroke and 39.5 mL in patients with right-sided stroke.

Patients with right MCA infarctions stated significantly more often to have lost their working ability compared to patients with left MCA infarctions (*p* < 0.05). Moreover, 32.4% of those with right-sided strokes and 17.6% of those with left-sided strokes reported loss or change of hobbies. Loss of driving ability was reported by 13.5% of right MCA infarction patients and 11.7% of left MCA infarction patients, as shown in Table 2. The mRS scores at follow-up ranged from 0 to 3, with a median of 1.

**Table 2:** Follow-up neuropsychological assessment.

Characteristics in neuropsychological assessment	Left-sided stroke	Right-sided stroke	*p*-value^†^
**Total patients included at follow-up, *n* (%)**	25 (47.2)	28 (52.8)	
**Age [years], mean (SD)**	65.3 (15.0)	63.5 (13.4)	
**SIS-16 questionnaire, mean (SD)^‡^**	70.7 (12.0)	72.0 (7.8)	
**SF-36 questionnaire, mean (SD), (+/−)^§^**			
Physical functioning	68.2 (31.2), (−)	72.3 (25.5), (−)	>0.1
Physical role functioning	59.8 (43.1), (−)	51.8 (44.6), (−)	>0.1
Bodily pain	76.0 (28.9), (+)	65.9 (30.3), (+)	>0.1
General health perceptions	59.7 (24.3), (+)	56.4 (19.0), (+)	>0.1
Vitality	57.5 (21.7), (−)	42.5 (21.6), (−)	<0.05
Social role functioning	77.0 (28.1), (−)	71.9 (24.9), (−)	>0.1
Emotional role functioning	59.7 (47.1), (−)	53.7 (48.1), (−)	>0.1
Mental health	72.3 (21.7), (+/−)	63.2 (19.4), (−)	>0.1
Physical component summary	45.1 (11.4), (+)	43.0 (9.9), (+)	>0.1
Mental component summary	47.4 (11.8), (−)	42.2 (10.6), (−)	>0.1
**General questions, *n* (%)^¶^**	34 (47.9)	37 (52.1)	
Loss of driving ability	4 (11.7)	5 (13.5)	>0.1
Loss of working ability	1 (2.9)	9 (24.3)	<0.05
Loss of hobbies	6 (17.6)	12 (32.4)	>0.1
Recurrent stroke	4 (11.7)	4 (10.8)	
Seizures	1 (2.9)	5 (13.5)	
Psychologic problems	5 (14.7)	7 (18.9)	

† SF-36: two-sided *p*-value according to independent sample *t*-test between the patient groups; general questions: two-sided *p*-value according to Pearson’s chi-squared test comparing the patient groups.

‡ Of a maximum of 80 points.

§ Compared to the reference group aged 61–70 years of the German normal control sample 1994: (+) higher score, (−) lower score, and (+/−) equivalent score.

¶ Percentage of those with telephone follow-up (left hemisphere *n* = 34, right hemisphere *n* = 37).

A total of 53 patients returned both the SIS-16 and SF-36 questionnaires (return rate >70%). The SIS-16 questionnaires showed overall similar results in patients with right- and left-hemispheric strokes, as shown in Table 2. The SF-36 questionnaire showed deficits in physical, social, and emotional role functioning in the patients with left- and right-hemispheric strokes compared to the age-specific norm of individuals aged 61–70 years. Significant differences between left- and right-hemispheric stroke were only found in the category of vitality (*p* < 0.05). No significant influence of age or follow-up duration was found on overall functional and health-related outcomes (SIS-16 and SF-36; *n* = 53) and loss of working ability (*n* = 31; 40 patients were already retired or not working at the time of stroke).

### Neuropsychological assessment

Of the 26 patients with stroke volume in the upper quartile selected for neuropsychological assessment, 3 denied consent and 8 could not be contacted. Of the remaining 15 patients, 66% had right-hemispheric strokes, and the mean age at follow-up was 62.8 ± 10.8 years. Mild cognitive impairment (score ≤25 on the Montreal Cognitive Assessment) was found in 9 of the 10 patients with right-hemispheric MCA infarction (mean score 20.5 ± 4.8) and in 2 of 5 patients with left-hemispheric MCA infarction (mean score 24.0 ± 2.8). No significant confounding was observed between age at stroke, follow-up duration, and the presence of cognitive impairment.

Alertness, which is defined as reaction time, was significantly reduced in patients with right- (*p* < 0.01) and left-hemispheric strokes (*p* < 0.05) compared to age-matched controls. All patients demonstrated normal inhibition ability and impulse control, as assessed by the Go/NoGo subtest (number of errors). The combination of both test results concluded that the patients’ intrinsic alertness was impaired. Furthermore, patients with right-sided brain lesions made a significantly higher number of omissions during the subtest divided attention in auditory, visual, and dual tasks compared to age-matched controls (*p* < 0.01). The results of the subtest visual scanning of patients with right-hemispheric stroke showed an average amount of omissions but significantly reduced reaction times in the two left columns, which indicates neglect to the left, as shown in Table 3.

**Table 3:** Neuropsychological assessment of the subcohort with stroke volumes in the upper quartile.

TAP-M subtest^†^	Left-sided stroke (*n* = 5)		Right-sided stroke (*n* = 10)	
	Mean (SD)	*p*-value^‡^	Mean (SD)	*p*-value^‡^
Alertness				
Median	39.2 (6.1)	<0.05	33.3 (4.6)	<0.001
Go/NoGo				
Errors	53.4 (12.0)	0.56	60.3 (17.1)	0.09
Visual field				
Median	43.6 (16.9)	0.45	41.0 (9.2)	<0.05
Omissions	55.2 (10.4)	0.33	48.6 (12.9)	0.29
Divided attention				
Auditory task: omissions	47.2 (6.3)	0.37	33.6 (9.7)	<0.001
Visual task: omissions	41.4 (12.6)	0.20	36.9 (9.6)	<0.01
Dual task: omissions	43.4 (11.9)	0.28	31.1 (8.7)	<0.001
Visual scanning				
Critical omissions	55.8 (6.6)	0.12	45.8 (10.8)	0.25
Non-critical median	35.2 (11.6)	<0.05	45.4 (12.8)	0.29
Column 1: median	40.0 (15.8)	0.23	38.4 (10.4)	<0.01
Column 1: omissions	54.2 (10.5)	0.42	44.5 (17.0)	0.33
Column 2: median	37.0 (11.6)	0.07	38.3 (13.8)	<0.05
Column 2: omissions	52.2 (8.0)	0.57	43.2 (16.6)	0.23

† All values are age-dependent standardized values (t-values).

‡ Two-sided *p*-values from one-sample *t*-test with *μ*_0_ = 50.

## Discussion

### Statement of principal findings

To the best of our knowledge, this is the first study to assess stroke lesion volumes and neuropsychological performance in patients with favorable outcomes (mRS 0-1) after acute ischemic stroke in the left or right middle cerebral artery territory and endovascular treatment. In this study population, patients with favorable outcomes had significantly larger stroke volumes when the stroke was located in the right hemisphere compared to the left hemisphere.

Additionally, our findings revealed neuropsychological and cognitive deficits, as well as reduced quality of life, in patients with large right-hemispheric strokes, despite being categorized as having a “favorable functional outcome” on the mRS.

Patients with right-hemispheric strokes showed significantly longer reaction times, made more errors, overlooked external stimuli, and had impaired divided attention compared to age-matched controls. A total of 90% of patients with right-hemispheric strokes had at least mild cognitive impairment in cognitive testing. We also observed a subclinical left-sided neglect in patients with right-sided lesions. Consistent with these findings, a significant proportion of patients with right MCA infarction reported their loss of working ability during telephone interviews, in contrast to patients with left MCA infarction.

In this study cohort, we combined assessment with the mRS, written questionnaires, telephone interviews, and neuropsychological tests, allowing sensitive measurement of neuropsychological dysfunction. When evaluating patients’ abilities, the mRS appears to be an appropriate scoring system for determining outcomes after left MCA infarction. However, the mRS misclassifies a considerable proportion of patients with right MCA infarction as favorable despite large infarct areas in brain imaging and substantial shortcomings in the quality of life and neuropsychological impairment. Our results are consistent with previous studies reporting that the mRS lacks sensitivity for cognitive and subtle neuropsychological impairments, especially after right-hemispheric stroke. While the mRS is valid and reliable as a global disability scale, it may overlook domain-specific impairments unless complemented by additional tools (6, 24).

Instruments such as the SIS-16 and SF-36 can provide a more nuanced understanding of a patient’s functional status and quality of life, although even these have limitations. In our cohort, both tests confirmed a similar outcome for post-stroke physical limitations for all patients and were thus not suitable for identifying the subclinical deficits. Significant ceiling effects for the SF-36 point to a lack of differentiation among various levels of low-level disabilities in social functioning (16, 23, 25).

Most importantly, the extensive neuropsychological assessment revealed significant differences in cognitive outcomes, including attention, processing speed, and executive function, after left and right MCA infarctions (9, 26). In accordance with previous studies, left neglect is often not detected by traditional screening tests and is therefore clinically unapparent. Dual tasks are necessary to sensitively reveal subclinical neglect in patients with right-hemispheric strokes (27). Our findings extend previous observations by demonstrating specific attentional deficits and subclinical neglect not captured by conventional outcome scales.

Methodological strengths of this study include highly accurate volume measurements performed by two independent, qualified raters, confirmed by the minimal inter-rater variability. We primarily used CT data, with MRI data used in seven cases where it was the only option available. Thus, CT and MRI data were treated as synonymous, even though the volume might differ substantially in some cases. Follow-up interviews to obtain the current mRS were conducted by telephone by a single qualified rater, and hence, the scores within our patient cohort showed a high level of validity (28). Nonetheless, patients’ self-reports, along with supplementary information provided by relatives, may not be completely reliable due to anosognosia. Stroke volume calculations could also be biased by the selection of patients undergoing thrombectomy, as thrombectomy is based on the NIHSS, which is biased toward dominant hemisphere lesions (29) and might therefore shift lesion size toward the right hemisphere. We conducted an extensive test battery to assess cognitive function thoroughly, even if only mildly impaired. However, some cognitive functions, such as attention, were more strongly represented than language and complex memory functions, which may overemphasize functions dominated by the right hemisphere. Furthermore, we may have introduced potential bias by restricting subsequent neuropsychological assessment to patients with lesion volumes >18 mL, since lesion volume is not the only determinant of the severity of cognitive symptoms.

Several factors known to influence post-stroke cognitive and psychological outcomes, such as metabolic parameters, cerebral small-vessel disease markers, comorbidities, and detailed information on post-acute rehabilitation strategies (30), were not systematically assessed in the registry and could not be included in the analysis. Although patients were interviewed about their medical history at follow-up, self-reported data were neither complete nor sufficiently reliable for further interpretation. While all patients received adequate early rehabilitation at the stroke unit of Frankfurt University Hospital, variability in access to community-based rehabilitation services, outpatient therapies, and family or social support after discharge may substantially modulate cognitive recovery and quality of life. These factors may partly explain interindividual differences in neuropsychological outcomes and represent an important limitation of the study. According to current literature, systematic screening for cognitive deficits, neglect, and mood disorders should be an integral part of post-stroke care, particularly in patients at risk of underrecognized deficits, such as those with large right-hemispheric infarction. Early identification should be followed by longitudinal monitoring and a multidisciplinary approach. Although evidence for specific cognitive rehabilitation strategies remains heterogeneous, targeted interventions—particularly for spatial and attentional deficits—have demonstrated benefits in selected patient populations, including those with subtle or subclinical neglect (31–35). Our findings support these recommendations by showing that clinically relevant neuropsychological deficits may persist in patients with right-hemispheric stroke despite favorable global functional outcomes, underscoring the need to extend outcome assessment beyond global disability scales.

Higher-order communication deficits commonly associated with right-hemispheric stroke, including impairments in affective prosody, pragmatic language use, and social discourse (36), were not explicitly assessed in our study. Consequently, right hemisphere-specific communication impairments may have gone unrecognized and could have contributed to persistent functional limitations not captured by the mRS. Given their potential impact on social participation, interpersonal communication, and return to work, these deficits represent an important target for future studies (37).

This study is limited by the lack of information on baseline mental health status prior to stroke, which may confound post-stroke neuropsychological outcomes. Prior research has shown that psychiatric disorders are associated with increased odds of ischemic stroke, suggesting that pre-stroke mental state may contribute both to stroke onset and to the cognitive and psychological state observed at follow-up (33, 38, 39). Notably, our results could only be confounded if baseline mental health were distributed differently by the hemisphere of stroke, which is not plausible. Nonetheless, future studies should include systematic assessments of pre-stroke mental health to differentiate pre-existing from stroke-related cognitive impairment.

The study was limited by its small cohort size due to patients lost to follow-up, which may have resulted in a selection bias toward engaged patients with potentially better outcomes and falsely good results in neuropsychological testing. Analyses addressing potential confounding factors revealed no association between age or follow-up duration and the assessed outcomes (questionnaires, cognitive impairment, and working ability). The limited sample size reduced the statistical power of our analyses and made it difficult to confidently extend our results to the broader population of stroke patients. However, the TAP-M test is designed to allow individual-level interpretation even in small clinical samples (21). Thus, we acknowledge that the subsample size for the TAP-M analysis (*n* = 15) was relatively small, which limited the statistical power and increased susceptibility to individual variation. However, we used age-standardized T-scores to minimize between-subject variability attributable to demographic factors. Furthermore, a one-sample *t*-test is appropriate for evaluating whether the group mean significantly differs from a known reference value, as in the case of standardized neuropsychological T-scores. However, not all variables met the assumption of normality. Consequently, the use of parametric tests must be interpreted with caution, as violations of normality can affect the validity of the results, particularly in small sample sizes. Furthermore, the time of follow-up varied between 1 and 4 years after stroke, limiting the comparability of patients, also due to a range of comorbidities. Although we corrected for important covariates, such as a patient’s age and gender, for the questionnaires and the TAP-M analysis, it was beyond the scope of this study to evaluate the effects of these factors. Surely, the clinical implications and practicability need further investigation and should be confirmed in larger clinical studies.

## Conclusion

In summary, our findings revealed neuropsychological and cognitive deficits, as well as reduced quality of life, in patients with right-hemispheric stroke despite being categorized as having a “good functional outcome” on the mRS. Overall, our results contribute to the growing body of evidence that outcome assessment after stroke must go beyond global disability scores to reflect the real-life impact of cognitive deficits—particularly those associated with right-hemispheric lesions. Future trials should consider our findings when evaluating treatment effectiveness in patients with right-hemispheric stroke and large lesion volumes, as neuropsychological impairments and cognitive deficits pose a substantial burden to both patients and their relatives.

## Data Availability

The data that support the findings of this study are not publicly available due to patient confidentiality. However, anonymized data may be shared upon reasonable request from the corresponding author, and only in part, to ensure compliance with privacy regulations. Requests will be reviewed by the institutional ethics committee to ensure that data sharing aligns with ethical guidelines.
